# Use of non-vitamin K antagonists oral anticoagulants in atrial fibrillation patients on dialysis

**DOI:** 10.3389/fcvm.2022.1005742

**Published:** 2022-09-13

**Authors:** Wenhao Li, Yanxia Zhou, Siqi Chen, Dewang Zeng, Haidong Zhang

**Affiliations:** ^1^Department of Nephrology, Affiliated Huadu Hospital, Southern Medical University (People's Hospital of Huadu District), Guangzhou, China; ^2^Department of Nephrology, Leliu Hospital Affiliated to Shunde Hospital of Guangzhou University of Chinese Medicine, Foshan, China; ^3^Department of Nephrology, Peking University Third Hospital, Beijing, China

**Keywords:** non-vitamin K antagonist oral anticoagulants, warfarin, atrial fibrillation, dialysis, meta-analysis

## Abstract

**Background:**

Non-vitamin K antagonist oral anticoagulants (NOACs) showed a benefit-risk profile superior to that of warfarin in atrial fibrillation (AF) patients with mild to moderate chronic kidney disease. However, the effectiveness and safety of NOACs in AF patients with end-stage renal disease (ESRD) on dialysis remain unclear. Therefore, we performed a meta-analysis regarding the effect of NOACs vs. warfarin in AF patients undergoing dialysis.

**Methods:**

A search of the Pubmed and EMBASE databases until November 2021 was performed. Adjusted risk ratios (RRs) and 95%confidence intervals (CIs) were pooled by a random-effects model with an inverse variance method.

**Results:**

Six studies involving 3,744 NOAC- and 26,973 warfarin- users were deemed to meet the criteria. In the pooled analysis, the use of mixed NOACs had similar incidences of effectiveness and safety outcomes compared with warfarin use. And factor Xa inhibitors (rivaroxaban or apixaban) did not have significantly better effectiveness than warfarin. For the safety outcomes, the use of factor Xa inhibitors was associated with a reduced risk of gastrointestinal bleeding (RR = 0.81, 95% CI 0.70–0.95), but not major bleeding and intracranial bleeding.

**Conclusion:**

Compared with warfarin, the use of NOACs, especially factor Xa inhibitors (rivaroxaban or apixaban), showed at least similar effectiveness and safety outcomes in AF patients on dialysis.

## Introduction

Patients with chronic kidney disease [CKD, especially end-stage renal disease (ESRD)] and atrial fibrillation (AF) are at higher risk of stroke or systemic thromboembolism (SSE) (1). Incidence of AF and worsening of CKD are linked with each other as they share several common risk factors (2). AF accelerates the progression to ESRD in patients with CKD, nearly doubles the mortality, and increases the stroke risk by ~6-fold in patients on dialysis (3), becoming one of the most important causes accounting for death among ESRD patients (4). An altered internal environment in CKD patients such as platelet dysfunction and hypercoagulability contributes to the development of AF in these patients. Dialysis is thought to be a trigger of AF in patients with ESRD as a high incidence of new-onset AF was observed after dialysis initiation (5).

AF is the most common indication for anticoagulation in patients with CKD (6). Warfarin has been used in patients with AF for decades (7). A prior meta-analysis showed that warfarin led to a much higher risk of bleeding in AF patients with ESRD on dialysis compared to those without anticoagulation (8). This might result from warfarin accumulation in these patients as CYP2C9 is downregulated in patients with ERSD (7, 9). And warfarin needs close monitoring of prothrombin time (10), deteriorates vascular calcification (11), and sometimes induces anticoagulant-related nephropathy (12).

NOACs [i.e., dabigatran (a direct thrombin inhibitor) and rivaroxaban, apixaban, and edoxaban (factor Xa inhibitors)] are alternatives for warfarin in AF-related stroke prevention. Several studies including different randomized clinical trials (13–16) and meta-analyses (17–19) have indicated a benefit-risk profile of NOACs superior to that of warfarin in patients with mild to moderate CKD, and other studies have demonstrated that there was no difference in bleeding rates between ESRD patients receiving apixaban and warfarin (20). One meta-analysis by Kuno et al. (21) investigated the efficacy of apixaban and warfarin in AF patients on dialysis and found they were not associated with a significant decrease in stroke and/or SSE. However, this analysis did not provide enough evidence as only 2 of 16 included studies in this meta-analysis investigated NOACs and the outcomes of dabigatran and rivaroxaban were limited to major bleeding events due to lack of data. Therefore, the effect of NOACs compared with warfarin in AF patients with ESRD on dialysis remains unclear. And the level of evidence and class of recommendation suggesting benefit or at least similar effect of NOACs compared with warfarin in this population was low and needed to be improved urgently. In this meta-analysis, we summarized the available data to compare the effectiveness and safety of NOACs vs. warfarin in this specific AF population.

## Methods

This meta-analysis was performed according to the guidance from the Cochrane Handbook for Systematic Reviews, the results of which were presented based on the PRISMA (Preferred Reporting Items for Systematic Reviews and Meta-analyses) items. Two reviewers (WH-L and YX-Z) independently performed the literature search, study selection, data abstraction, quality assessment, and data analysis. Disagreements were resolved by discussion between two reviewers, or consultation with the corresponding authors.

### Inclusion and exclusion criteria

We included randomized controlled trials (RCTs) or observational cohort studies if they compared at least one of the effectiveness and safety outcomes of NOACs (dabigatran, rivaroxaban, apixaban, or edoxaban) vs. warfarin in AF patients with ESRD on dialysis (hemodialysis or peritoneal dialysis). The effectiveness outcomes were a composite of SSE, ischemic stroke, and all-cause death, whereas the safety outcomes were major bleeding, intracranial bleeding, and gastrointestinal bleeding. The definitions of the studied outcomes were applied that were reported in the originally included studies. We excluded studies focusing on AF patients with cardioversion, ablation, or left-atrial appendage occluder. We also excluded studies with a sample size of <100. Certain publication types were excluded (e.g., reviews, comments, case reports, case series, letters, editorials, and meeting abstracts) due to insufficient data.

### Literature search

We systematically searched the PubMed and Embase databases until November 7, 2021, for identifying studies about the effectiveness and safety of NOACs compared with warfarin in AF patients with ESRD on dialysis. The search terms combined with “AND” were applied as follows: (1) “atrial fibrillation”, (2) “dialysis” OR “hemodialysis” OR “peritoneal dialysis” OR “end-stage kidney disease” OR “end-stage renal disease” OR “advanced renal disease”, (3) “vitamin K antagonist” OR “warfarin”, (4) “non-vitamin K antagonist oral anticoagulant” OR “direct oral anticoagulant” OR “novel oral anticoagulant” OR “NOAC” OR “DOAC” OR “dabigatran” OR “rivaroxaban” OR “apixaban” OR “edoxaban”. The detailed search strategies of this meta-analysis are presented in Supplementary Table 1. No linguistic restrictions were applied in the literature search.

### Study screenings and data abstraction

We first screened the titles and abstracts of the retrieved studies, and subsequently read the full texts of the potential studies. Eligible studies would be chosen based on the pre-defined inclusion criteria. The following information of the included studies was collected: first author, year of publication, study design, data source and study period, patient characteristics (study population, sample size, age, and sex), type and dosage of NOACs, follow-up time, and the effectiveness and safety outcomes.

### Study quality assessment

We assessed the bias risk of RCTs using the Cochrane Collaboration's tool on the selection bias, performance bias, detection bias, attrition bias, reporting bias, and other biases. For each domain of this tool, the level of the bias risk was scored as “low,” “unclear,” or “high” risk. In addition, the Newcastle-Ottawa Scale (NOS) tool was used to assess the quality of the observational cohort studies. The NOS tool had three domains with a total of nine points: the selection of cohorts (0–4 points), the comparability of cohorts (0–2 points), and the assessment of the outcome (0–3 points). In this meta-analysis, studies with an NOS of <6 points were defined as a low quality (22, 23).

### Statistical analysis

The statistical heterogeneity across the included studies was assessed using the *P*-value of the Cochrane *Q*-test and the *I*^2^ statistic, where a *P*-value of < 0.10 in the Cochrane *Q*-test or an *I*^2^-value of > 50% suggested significant heterogeneity. For the included studies reporting unadjusted effect estimates, we collected the sample size and the number of events in the warfarin- or NOAC- groups and then calculated the unadjusted event rates between the two groups, which were expressed as the odds ratios. For those studies reporting adjusted data with multiple models, we applied the most adjusted risk ratios (RRs) and 95%confidence intervals (CIs). In the main pooled analysis, the effect estimates were converted to the natural logarithms and standard errors, which were pooled by a DerSimonian and Laird random-effects model with an inverse variance method. In the secondary analysis, since the use of dabigatran had limited evidence in AF patients with ESRD on dialysis, we excluded the data of dabigatran and re-performed the meta-analysis. The subgroup analysis was performed based on the type and dosage of NOACs. In the sensitivity analysis, we re-performed the above-mentioned analysis using a fixed-effects model. We also excluded the unadjusted data or the data of RCT in the pooled analysis. According to the Cochrane book, we did not perform the publication bias analysis if the number of the included studies was <10.

All the statistical analyses of this meta-analysis were performed using the Review Manager version 5.4 software (the Cochrane Collaboration 2014, Nordic Cochrane Center Copenhagen, Denmark; https://community.cochrane.org/). In this study, a *P*-value of < 0.05 was considered statistically significant.

## Results

### Study selection

The flow chart of the literature retrieval is presented in Supplementary Figure 1. A total of 736 retrieved studies were retrieved in the Pubmed and Embase databases. After the first phase of the title- and abstract- screenings, 11 remaining studies were potentially available, which were assessed by the full-text screenings. Subsequently, we excluded 5 studies because (1) warfarin was not the reference (*n* = 2) (24, 25); (2) study focused on ESRD patients with AF or venous thromboembolism (*n* = 1) (20); (3) study included a sample size of <100 in the analysis (*n* = 1) (26); and (4) study with an overlapping data (*n* = 1) (27). Finally, a total of 6 studies (1 RCT and 5 observational cohorts) (28–33) involving 3,744 NOAC- and 26,973 warfarin- users were included in this meta-analysis.

### Baseline characteristics of the included studies

Table 1 shows the baseline characteristics of the included studies. In hemodialysis patients with AF, a prior RCT in 2020 published by De Vriese et al. (27) compared the primary endpoint of the progression of cardio-aortic calcium deposits among warfarin, rivaroxaban, and rivaroxaban plus vitamin K2 with a follow-up of 18 months. In this trial, they additionally followed for at least 18 months and compared the effectiveness and safety outcomes of rivaroxaban compared with warfarin (29). Although the studies by See et al. (28) and Lin et al. (31) used the same data source of Taiwan's National Health Insurance Research Database, See et al. (28) reported a mixed type of NOACs including dabigatran, rivaroxaban, apixaban, and edoxaban, whereas Lin et al. (31) focused on the use of rivaroxaban. Therefore, the data of See et al. (28) and Lin et al. (31) were applied in different parts of our meta-analysis. Chan et al. (33) assessed the effect of dabigatran and rivaroxaban separately, whereas Ionescu et al. (30) and Siontis et al. (32) focused on the use of apixaban. The administrated dosages of different NOACs in patients in the included studies are listed in Table 1. For the quality assessment, the Valkyrie study by De Vriese et al. (29) had a low risk of bias, details of the assessment are presented in Supplementary Table 2. All 5 observational cohorts had an acceptable quality with the NOS tool of ≥6 points.

**Table 1:** Baseline characteristics of the included studies.

**References**	**Database source**	**Study design**	**AF patients on dialysis**	**Age (y)/Sex**	**Sample size**	**NOAC dose**	**Follow-up (y)**	**Quality assessment**
De Vriese et al. (29)	The Valkyrie study	RCT	Patients on chronic hemodialysis	71.5–84.3/both	Rivaroxaban (*n* = 88); Warfarin (*n* = 44)	Rivaroxaban 10 mg QD (100%)	1.88	Low risk of bias
See et al. (28)	Taiwan's National Health Insurance Research Database; 06/2012–12/2017	Retrospective cohort	ESRD patients on chronic dialysis	74.8/both	Dabigatran (*n* = 150); Rivaroxaban (*n* = 224); Apixaban (*n* = 72); Edoxaban (*n* = 17); Warfarin (*n* = 8,064)	Dabigatran 110 mg BID (92%); Rivaroxaban 15/10 mg QD (96%); Apixaban 2.5 mg BID (82%); Edoxaban 30 mg BID (89%)	NA	NOS = 7 points
Ionescu et al. (30)	Academic healthcare system in Southeast Michigan, USA	Retrospective cohort	Patients on chronic hemodialysis	67.2/both	Apixaban (*n* = 144); Warfarin (*n* = 563)	Apixaban 5 mg BID (36%) and 2.5 mg BID (64%)	NA	NOS = 6 points
Lin et al. (31)	Taiwan's National Health Insurance Research Database; 02/2013–09/2017	Retrospective cohort	ESRD patients on regular dialysis	69.0/both	Rivaroxaban (*n* = 173); Warfarin (*n* = 3,185)	Rivaroxaban 20 mg QD (10.4%), 15 mg QD (38.7%), and 10 mg QD (50.8%)	1.59	NOS = 7 points
Siontis et al. (32)	Medicare beneficiaries included in the United States Renal Data System; 10/2010–12/2015	Retrospective cohort	ESRD patients on peritoneal dialysis or hemodialysis	68.2/both	Apixaban (*n* = 2,351); Warfarin (*n* = 7,053)	Apixaban 5 mg BID (44%) and 2.5 mg BID (56%)	NA	NOS = 8 points
Chan et al. (33)	Fresenius Medical Care North America ESRD database; 10/2010–10/2014	Retrospective cohort	Patients on hemodialysis	70.4/both	Dabigatran (*n* = 281); Rivaroxaban (*n* = 244); Warfarin (*n* = 8,064)	Dabigatran 150 mg BID (15.3%) and 75 mg BID (84.7%); Rivaroxaban 20 mg QD (32.1%) and 15 mg QD (67.8%)	2.0	NOS = 8 points

AF, atrial fibrillation; RCT, Randomized Controlled Trial; ESRD, end-stage renal disease; NOACs, non-vitamin K oral anticoagulants; NOS, Newcastle-Ottawa Scale; NA, not available.

### Effect of mixed NOACs vs. warfarin in dialysis patients with AF

In the main pooled analysis, our results based on the random-effects model showed that compared with warfarin use, the use of NOACs was not significantly associated with the effectiveness outcomes including SSE (RR = 0.95, 95% CI 0.68–1.31; *P* = 0.74; *I*^2^ = 51%), ischemic stroke (RR = 0.93, 95% CI 0.55–1.60; *P* = 0.80; *I*^2^ = 41%), and all-cause death (RR = 0.84, 95% CI 0.71–1.00; *P* = 0.05; *I*^2^ = 0%), and safety outcomes including major bleeding (RR = 0.96, 95% CI 0.65–1.43; *P* = 0.85; *I*^2^ = 89%), intracranial bleeding (RR = 0.75, 95% CI 0.50–1.14; *P* = 0.18; *I*^2^ = 0%), and gastrointestinal bleeding (RR = 0.87, 95% CI 0.74–1.01; *P* = 0.07; *I*^2^ = 0%) (Supplementary Figures 2, 3).

### Effect of factor Xa inhibitors vs. warfarin in dialysis patients with AF

**Figure 1:**
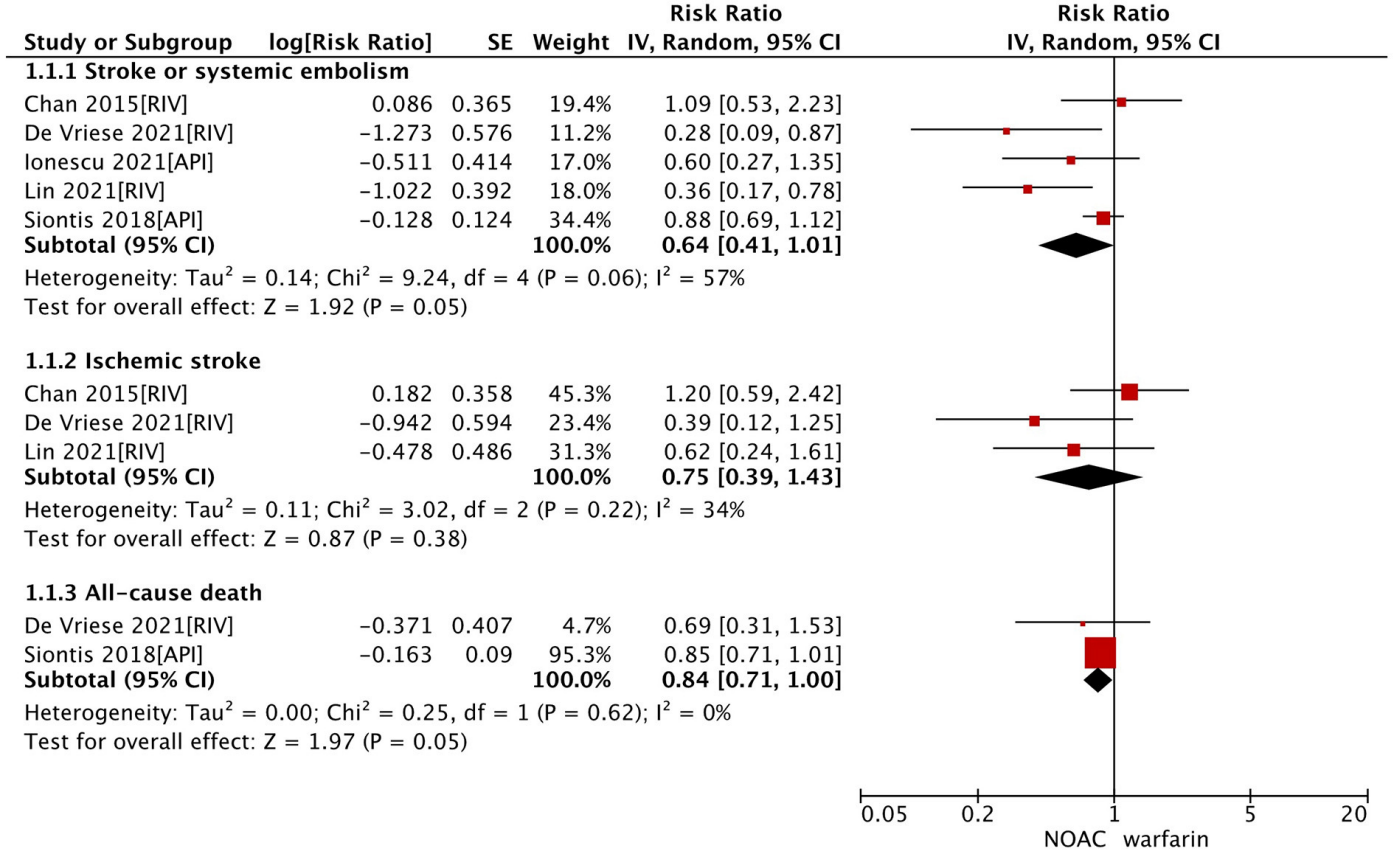
Effectiveness outcomes of NOACs vs. warfarin in dialysis patients with atrial fibrillation. CI, confidence interval; SE, standard error; IV, inverse of the variance.

**Figure 2:**
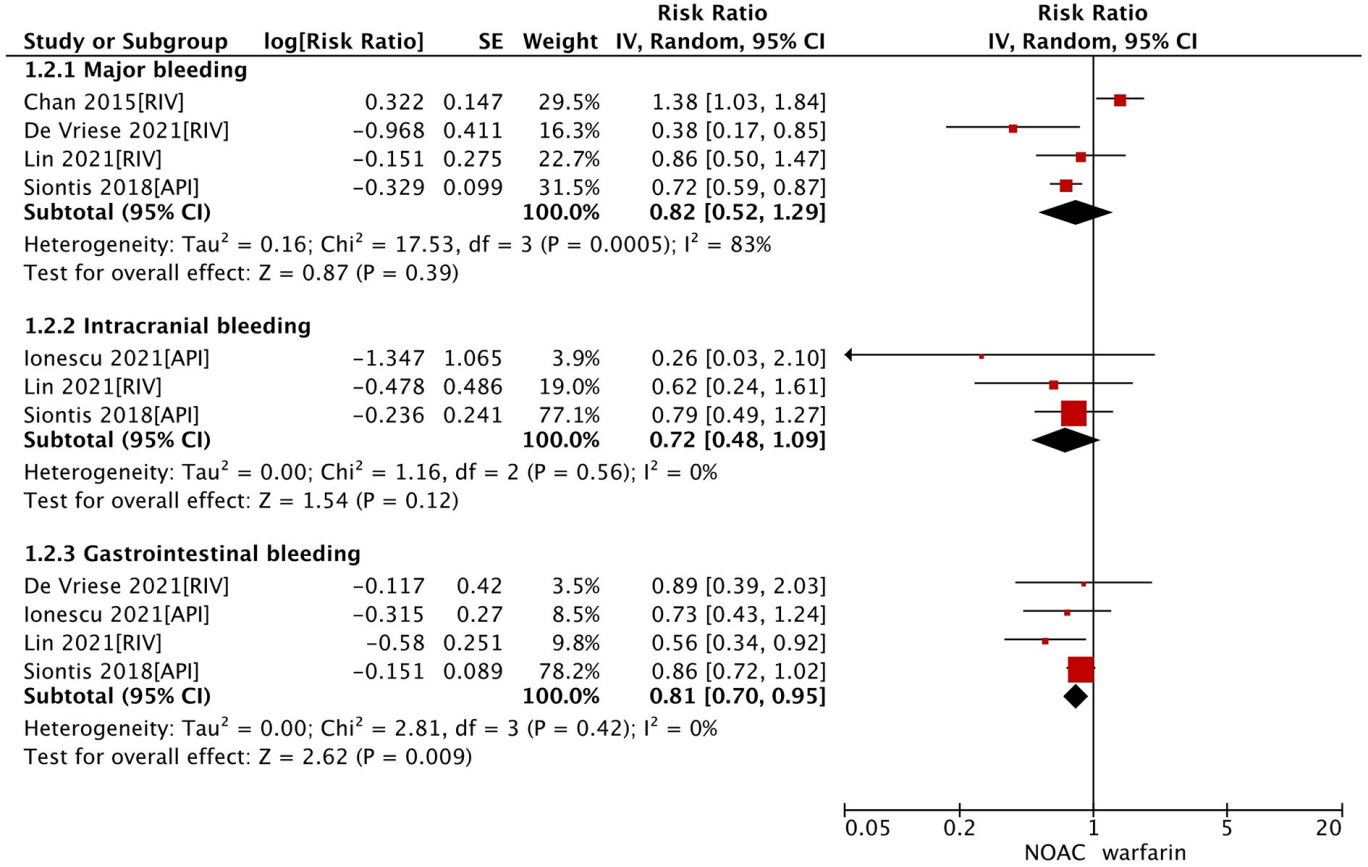
Safety outcomes of factor Xa inhibitors (rivaroxaban or apixaban) vs. warfarin in dialysis patients with atrial fibrillation. CI, confidence interval; SE, standard error; IV, inverse of the variance.

In the secondary analysis, we excluded studies with the data of dabigatran (28, 33) and assessed the effect of factor Xa inhibitors (rivaroxaban or apixaban) compared with warfarin in dialysis patients with AF. As shown in Table 2, our pooled results based on the random-effects model showed that the use of factor Xa inhibitors did not alter the risk of SSE (RR = 0.64, 95% CI 0.41–1.01; *P* = 0.05; *I*^2^ = 57%) and risk of all-cause death (RR = 0.84, 95% CI 0.71–1.00; *P* = 0.05; *I*^2^ = 0%) significantly compared to warfarin (Figure 1). For the safety outcomes, compared with warfarin use, the use of factor Xa inhibitors was associated with a decreased risk of gastrointestinal bleeding (RR = 0.81, 95% CI 0.70–0.95; *P* = 0.009; *I*^2^ = 0%), but there were no differences in major bleeding (RR = 0.82, 95% CI 0.52–1.29; *P* = 0.39; *I*^2^ = 83%) and intracranial bleeding (RR = 0.72, 95% CI 0.48–1.09; *P* = 0.12; *I*^2^ = 0%) between the two groups (Figure 2).

**Table 2:** Effectiveness and safety outcomes between NOACs and warfarin in dialysis patients with AF.

	**Stroke or systemic embolism**	**Ischemic stroke**	**All-cause death**	**Major bleeding**	**Intracranial bleeding**	**Gastrointestinal bleeding**
**Main analysis: mixed NOACs**						
No. of effect estimates	6	4	2	5	3	4
RRs and 95% CIs	0.95 (0.68, 1.31)	0.93 (0.55, 1.60)	0.84 (0.71, 1.00)	0.96 (0.65, 1.43)	0.75 (0.50, 1.14)	0.87 (0.74, 1.01)
*P-*value	0.74	0.8	0.05	0.85	0.18	0.07
*I*^2^ statistic	51%	41%	0%	89%	0%	0%
**Secondary analysis: factor Xa inhibitors**						
No. of effect estimates	4	3	2	4	3	4
RRs and 95% CIs	0.64 (0.41, 1.01)	0.75 (0.39, 1.43)	0.84 (0.71, 1.00)	0.82 (0.52, 1.29)	0.72 (0.48, 1.09)	0.81 (0.70, 0.95)
*P-*value	0.05	0.38	0.05	0.39	0.12	0.009
*I*^2^ statistic	57%	34%	0%	83%	0%	0%
**Subgroup analysis**						
**1) Rivaroxaban**						
No. of effect estimates	3	2	-	3	1	2
RRs and 95% CIs	0.51 (0.22, 1.20)	0.76 (0.26, 2.23)	-	0.84 (0.43, 1.63)	0.62 (0.24, 1.61)	0.63 (0.41, 0.96)
**Apixaban**						
No. of effect estimates	2	-	-	1	2	2
RRs and 95% CIs	0.85 (0.68, 1.08)	-	-	0.72 (0.59, 0.87)	0.77 (0.49, 1.22)	1.44 (0.43, 4.77)
**2) High dose of NOACs**						
No. of effect estimates	1	-	-	3	-	-
RRs and 95% CIs	0.64 (0.42, 0.97)	-	-	1.57 (0.63, 3.90)	-	-
**Low dose of NOACs**						
No. of effect estimates	3	-	-	5	-	-
RRs and 95% CIs	0.51 (0.18, 1.44)	-	-	0.85 (0.56, 1.29)	-	-
**Sensitivity analysis**						
**1) Only included adjusted data**						
No. of effect estimates	2	1	1	4	2	2
RRs and 95% CIs	0.97 (0.73, 1.29)	0.62 (0.24, 1.61)	0.85 (0.71, 1.01)	1.10 (0.74, 1.63)	0.79 (0.51, 1.21)	0.88 (0.75, 1.04)
*P-*value	0.83	-	-	0.65	0.27	0.13
*I*^2^ statistic	30%	-	-	90%	0%	0%
**2) Deleting the data of RCT**						
No. of effect estimates	5	3	1	4	3	3
RRs and 95% CIs	1.02 (0.79, 1.32)	1.14 (0.74, 1.77)	0.85 (0.71, 1.01)	1.10 (0.74, 1.63)	0.75 (0.50, 1.14)	0.87 (0.74, 1.01)
*P-*value	0.89	0.55	-	0.65	0.18	0.07
*I*^2^ statistic	28%	8%	-	90%	0%	0%
**3) Re-analysis with a fixed-effects model**						
No. of effect estimates	5	4	2	5	3	4
RRs and 95% CIs	0.95 (0.79, 1.14)	1.02 (0.69, 1.51)	0.84 (0.71, 1.00)	1.05 (0.93, 1.18)	0.75 (0.50, 1.14)	0.87 (0.74, 1.01)
*P-*value	0.58	0.92	0.05	0.46	0.18	0.07
*I*^2^ statistic	51%	41%	0%	89%	0%	0%

AF, atrial fibrillation; RR, risk ratio; CI, confidence interval; NOACs, non-vitamin K antagonist oral anticoagulants; RCT, Randomized Controlled Trial.

### Subgroup analysis and sensitivity analysis

In terms of SSE and major bleeding, the subgroup analysis based on the NOAC type showed that there were no interactions between rivaroxaban vs. apixaban. In addition, there were also no significant differences in SSE and major bleeding between the high vs. low dose of NOACs (Table 2).

As shown in Table 2, for the effectiveness and safety outcomes, re-analysis with the fixed-effects model showed similar results as the main pooled analysis with the random-effects model. In addition, we also observed similar results as the main analysis when excluding the studies with unadjusted data or excluding the RCT of De Vriese et al. (29).

## Discussion

Our current study indicated that the use of mixed NOACs had similar incidences of effectiveness and safety outcomes compared with warfarin use in AF patients with ESRD on dialysis. Specifically, the use of factor Xa inhibitors (rivaroxaban or apixaban) had a decreased risk of gastrointestinal bleeding compared with warfarin use. This specific effect might result from decreased absorption function of the gastrointestinal tract in patients with uremia. In uremia, the absorption of NOACs becomes slower and a larger amount of NOACs accumulates in the gastrointestinal tract. This process might be even more obvious in rivaroxaban as the bioavailability of it increases if it is taken together with food (1). Such an assumption could be proved by a mouse model in the future. Overall, the use of NOACs, especially factor Xa inhibitors (rivaroxaban or apixaban), showed at least similar effects compared with warfarin use in dialysis patients with AF.

We queried the outcomes of the prior meta-analysis by Kuno et al. (21) as only 2 included studies investigated NOACs and the sample size is relatively small. In addition, a similar study conducted by Chen et al. (9) summarized that the use of rivaroxaban or apixaban might be associated with reduced risks of all-cause death and gastrointestinal bleeding in AF patients with stage 4–5 CKD or on dialysis. And another meta-analysis by See et al. (28) suggested similar effectiveness and safety outcomes between NOACs and warfarin among AF patients with stage 4–5 CKD on dialysis. These two studies by Chen et al. (9) and See et al. (28) did not focus on the AF patients with ESRD on dialysis and thus the effect of NOACs in this specific population remained debatable for us to investigate. However, the data we summarized showed the use of factor Xa inhibitors (rivaroxaban or apixaban) did not alter the risks of SSE and all-cause death significantly compared to warfarin as both confidence intervals cross one (95% CI 0.41–1.01 for risks of SSE and 95% CI 0.71–1.00 for all-cause mortality, respectively). We hoped future observational studies or RCTs could focus on hazard ratio and bring a new answer to the question of whether NOACs could lengthen the survival time of AF patients on dialysis or not. In terms of gastrointestinal bleeding, a previous meta-analysis by Burr et al. (34) demonstrated that factor Xa inhibitors were associated with a reduced risk of all severities of gastrointestinal bleeding compared with warfarin, but not specifically in AF patients with ESRD on dialysis. We remedied this weakness and the summarized data indicated that in this specific population the use of factor Xa inhibitors was associated with a decreased risk of gastrointestinal bleeding. Our findings support the FDA's recommendation of rivaroxaban and apixaban in patients with ESRD and AF (2). While European guideline recommended patients on dialysis as well as patients with severe renal dysfunction (CrCl < 15 mL/min) should refrain from NOACs use (35), our study supported that factor Xa inhibitors (apixaban and rivaroxaban) in AF patients with ESRD on dialysis is at least not a worse choice compared to warfarin. In fact, anticoagulation in this specific population must be individualized through a multidisciplinary approach.

Although apixaban and rivaroxaban show potential advantages over warfarin, the dosage of these drugs for a better effectiveness and safety outcome in AF patients with ESRD on dialysis remains unclear. In one of our included studies, Siontis et al. (32) compared the different roles of different dosages of apixaban in this population, suggesting that a standard dose (5 mg twice daily) is associated with lower risks of SSE and death, whereas a low dose (2.5 mg twice daily) presents a lower risk of major bleeding. Kuno et al. (21) reported that apixaban 5 mg twice daily was associated with a lower risk of mortality for patients with AF on long-term dialysis compared to other treatments (apixaban 2.5 mg twice daily or no anticoagulants). Because of this uncertain benefit-to-harm ratio of NOACs in AF patients on dialysis, the nephrological guidelines KDIGO (Kidney Disease: Improving Global Outcomes) still recommend warfarin as the first choice drug for anticoagulation (36).

The effectiveness and safety outcomes of NOACs seemed to improve after we excluded the data of dabigatran, suggesting low effectiveness and safety of dabigatran in AF patients with ESRD on dialysis. This could be explained by the pharmacokinetic and pharmacodynamic characteristics of dabigatran. First, the effect of dabigatran might be reduced in hemodialysis patients as 50–60% of dabigatran is dialyzable (1). Second, clinical use of dabigatran shortly after its approval in the United States showed high rates of major and non-major bleeding in patients with hemodialysis (37), this might result from the high renal clearance rate of dabigatran (~80%) (38) and accumulation of dabigatran in patients with severe renal impairment (a 6.3-fold higher AUC in these patients) (39). Therefore, rivaroxaban, apixaban, and edoxaban (but not dabigatran) are approved in Europe for use in patients with severe CKD, with a reduced dose regimen. In view of individual pharmacokinetics, edoxaban might be another NOAC with clinical effectiveness and safety comparable to apixaban and rivaroxaban as hemodialysis only led to a minor decrease in a total exposure of edoxaban and hemodialysis did not affect edoxaban's concentration in 24 h (40). However, the effectiveness and safety of edoxaban in AF patients with ESRD on dialysis remains unclear due to limited data. Only one RCT by Bohula et al. (14) and one observational study by Yu et al. (41) reported edoxaban was associated with reduced bleeding risk in patients with GFR 30–50 ml/min, respectively. Further studies on the data of edoxaban with a larger sample size might help establish its clinical effect in AF patients with ESRD on dialysis.

### Limitations

Our current meta-analysis still had several limitations. First, it's still insufficient to make recommendations of NOACs for AF patients on dialysis based on our study as we only included 1 RCT and 5 observational cohorts. More data from large RCTs are considered to be a preferable way to bring clarity to this question. And the all-cause death endpoint was evaluated in only 2 of the 6 meta-analyzed studies. Second, although we performed the subgroup analysis based on the type and dosage of NOACs, dosage variability of NOACs in our study showed no difference in SSE and major bleeding, further scrutinized analysis is restricted given the limited patients number. The results of subgroup analyses should be interpreted cautiously. The data of dabigatran could not be assessed in the subgroup analysis because only one study by Chan et al. (33) studied the use of dabigatran vs. warfarin. In addition, comparative effectiveness and safety outcomes of edoxaban compared with warfarin were not assessed because of the limited data. Third, according to the Cochrane handbook, the publication bias was not formally assessed when the number of included studies was <10. As such, the results of publication bias should be interpreted cautiously and further assessed. Fourth, we pooled the unadjusted and adjusted data in the main analysis. Although we observed similar findings as the main analysis when only including the studies with adjusted data, the potential unmeasured confounders still existed. Fifth, ESRD patients on peritoneal dialysis and hemodialysis were not separately analyzed in our present study due to the limiting data. Finally, this review was not pre-registered online.

## Conclusion

The use of NOACs, especially factor Xa inhibitors (rivaroxaban or apixaban), showed at least similar effectiveness and safety outcomes compared with warfarin use in dialysis patients with AF.

## Data availability statement

The original contributions presented in the study are included in the article/Supplementary material, further inquiries can be directed to the corresponding authors.

## Author contributions

All authors listed have made a substantial, direct, and intellectual contribution to the work and approved it for publication.

## Funding

This study was supported by the Intramural Research Program of People's Hospital of Huadu District (2021C03); the Program of Huadu District Science and Technology (20-HDWS-033); Guangzhou Medical Key Subject Construction Project of China (2021-2023).

## Conflict of interest

The authors declare that the research was conducted in the absence of any commercial or financial relationships that could be construed as a potential conflict of interest.

## Publisher's note

All claims expressed in this article are solely those of the authors and do not necessarily represent those of their affiliated organizations, or those of the publisher, the editors and the reviewers. Any product that may be evaluated in this article, or claim that may be made by its manufacturer, is not guaranteed or endorsed by the publisher.
