# Supplementary material for: Use of non-vitamin K antagonists oral anticoagulants in atrial fibrillation patients on dialysis
Source: Front Cardiovasc Med. 2022 Sep 13;9:1005742. doi: 10.3389/fcvm.2022.1005742 (PMC9513185; doi:10.3389/fcvm.2022.1005742)
Supplement: Supplementary file 1 [file Data_Sheet_1.docx]

**Supplementary Table 1. The search strategies of this meta-analysis**

|  | **Search terms** | **Pubmed** | **Embase** |
| --- | --- | --- | --- |
| #1 | atrial fibrillation | 93992 | 194078 |
| #2 | dialysis | 203148 | 241646 |
| #3 | hemodialysis | 164396 | 167213 |
| #4 | peritoneal dialysis | 34517 | 52249 |
| #5 | end-stage kidney disease | 130255 | 7437 |
| #6 | end-stage renal disease | 136202 | 74733 |
| #7 | advanced renal disease | 33589 | 358 |
| #9 | #2 OR #3 OR #4 OR #5 OR #6 OR #7 | 311589 | 356263 |
| #10 | novel oral anticoagulant | 2710 | 700 |
| #11 | direct oral anticoagulant | 7522 | 2194 |
| #12 | non-vitamin K antagonist oral anticoagulant | 1511 | 417 |
| #13 | NOAC | 1611 | 3728 |
| #14 | DOAC | 2049 | 4125 |
| #15 | dabigatran | 6070 | 18628 |
| #16 | rivaroxaban | 6961 | 22362 |
| #17 | apixaban | 4539 | 16065 |
| #18 | edoxaban | 1871 | 6252 |
| #19 | #10 OR #11 OR #12 OR #13 OR #14 OR #15 OR #16 OR #17 OR #18 | 18242 | 37943 |
| #20 | vitamin K antagonist | 9488 | 5383 |
| #21 | warfarin | 32068 | 103162 |
| #22 | #20 OR #21 | 38875 | 106266 |
| #23 | #1 AND #9 AND #19 AND #22 | 258 | 478 |

**Supplementary Table 2. Risk assessment of the included RCT**

| **Contents for risk assessment** | **Assessment justification** | **Ratings** |
| --- | --- | --- |
| **De Vriese-2021** |  |  |
| Random sequence generation (selection bias) | A central, web-based,  computerized randomization system applying a  minimization algorithm randomly assigned participants  (1:1) to initiate and stop anticoagulant treatment | Low risk |
| Allocation concealment (selection bias) | The unique study identification number was allocated to every participants who were administrated to oral anticoagulants | Low risk |
| Blinding of participants and personnel (performance bias) | Participants, clinicians and local investigators knew the treatment assignments, but participant identity, treatment allocation,  and drug use was unknown to event adjudicators | Low risk |
| Blinding of outcome assessment (detection bias) | Reports of every outcome events were performed by internal assessor -- one medically trained clinical research fellow, and investigators rated dependence and quality of life was evaluated by modified Rankin Scale and EQ-5D-5L, respectively | Low risk |
| Incomplete outcome data (attrition bias) | Completeness of follow-up was performed by follow-up  questionnaire at each planned interval after randomization | Low risk |
| Selective reporting (reporting bias) | NS | UNCLEAR |
| Other risk biases | NS | UNCLEAR |


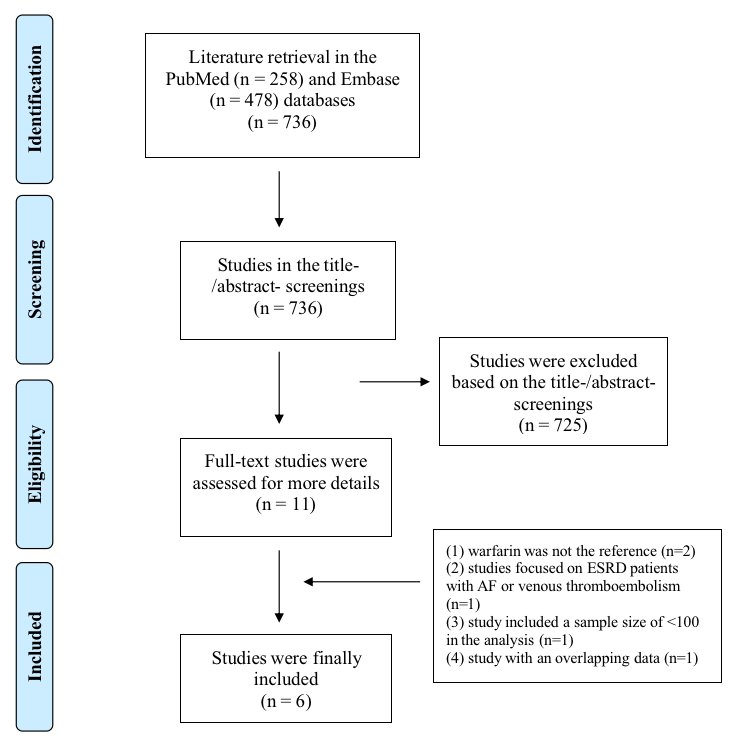


**Supplementary Figure 1. The flow chart of document retrieval in this meta-analysis**


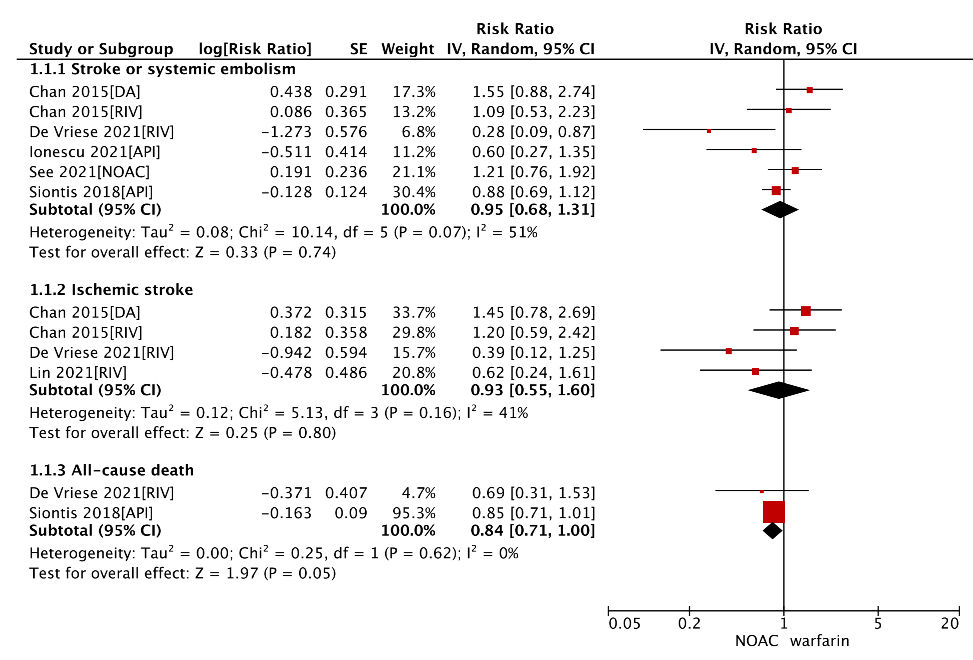


**Supplementary Figure 2. Effectiveness outcomes of mixed NOACs versus warfarin in dialysis patients with AF**


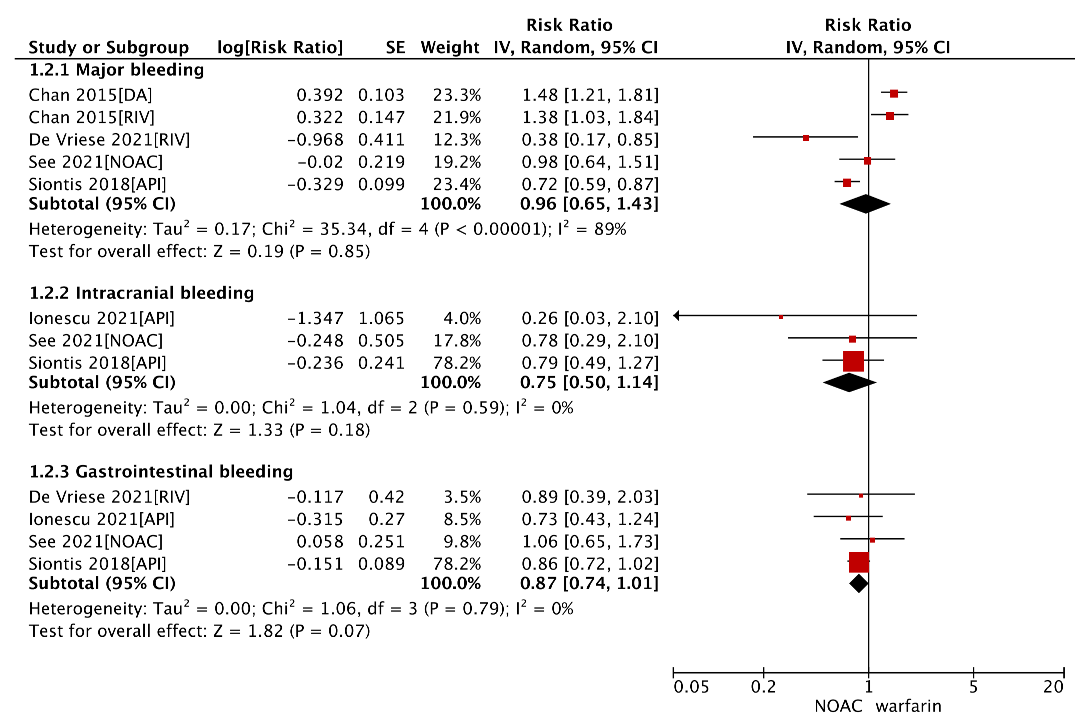


**Supplementary Figure 3. Safety outcomes of mixed NOACs versus warfarin in dialysis patients with AF**
